# Supplementary material for: Hydrogen bond symmetrisation in D2O ice observed by neutron diffraction
Source: Nat Commun. 2024 Jun 27;15:5100. doi: 10.1038/s41467-024-48932-8 (PMC11211428; doi:10.1038/s41467-024-48932-8)
Supplement: Supplementary file 1 — Supplementary Information [file 41467_2024_48932_MOESM1_ESM.pdf]

Supplementary Information

Hydrogen bond symmetrisation in D_2O ice observed by neutron diffraction

Kazuki Komatsu[1,*], Takanori Hattori[2], Stefan Klotz[3], Shinichi Machida[4], Keishiro Yamashita[1,†], Hayate Ito[1], Hiroki Kobayashi[1], Tetsuo Irifune[5], Toru Shinmei[5], Asami Sano-Furukawa[2], Hiroyuki Kagi[1]

[1]Geochemical Research Center, Graduate School of Science, The University of Tokyo, Hongo 7-3-1, Bunkyo-ku, Tokyo, 113-0033, Japan

[2]J-PARC Center, Japan Atomic Energy Agency, Tokai, Naka, Ibaraki 319-1195, Japan

[3]Institut de Minéralogie, de Physique des Matériaux et de Cosmochimie, CNRS UMR 7590, Sorbonne Université, F-75252 Paris, France

[4]Neutron Science and Technology Center, CROSS, Tokai, Naka, Ibaraki 319-1106, Japan

[5]Geodynamics Research Center, Ehime University, 2-5 Bunkyo-cho, Matsuyama, Ehime 790-8577, Japan

[*]Corresponding author:

e-mail : kom@eqchem.s.u-tokyo.ac.jp

[†]Present address:

Institute of Physical Chemistry, University of Innsbruck, A-6020 Innsbruck, Austria

Supplementary Information

1. Detailed experimental conditions and results.

Detailed experimental conditions for each run are presented in Supplementary Table 1. The obtained diffraction intensities normalised with attenuation corrections for #1 to #7, which is based on the treatment described in Supplementary Information 4 (attenuation corrections), are shown in Supplementary Figures 1 to 7, respectively. Note that the reported diffraction data for #1 and #2 in Komatsu et al.[1] are non-corrected for wavelength dependent factors such as incident neutron profiles, detector efficiency, attenuation corrections etc., whereas diffraction patterns shown here are all corrected. The results of peak fitting and the Rietveld analyses for #1 to #7 are shown in Supplementary Tables 2 to 8, respectively.

Supplementary Table 1. Anvil, gasket, diffraction geometries, and temperature path for sample preparation for respective experiments. See text for details of anvil shape, diffraction geometry (t/a : through anvils, t/g: through gasket) and pressure-temperature path

Exp ID		Anvil			Gasket			
Run#	YearMonth_#	Shape	Culet (mm)	Dip diameter (mm)	Material	Initial thickness (mm)	Thickness after pre-indentation (mm)	Initial hole diameter (mm)
#1	2020Jan_01	Cupped	1.0	0.5	SUS301	0.260	0.21	0.40
#2	2020Jan_02	Flat	1.0	-	SUS301	0.260	0.14	0.40
#3	2021Mar_01	Cupped	0.5	0.3	SUS301	0.260	0.20	0.30
#4	2022Apr_01	Cupped	1.0	0.5	TiZr	0.350	0.27	0.50
#5	2022Apr_03	Cupped	0.5	0.3	SUS301	0.260	0.22	0.30
#6	2023Apr_03	Cupped	0.5	0.3	SUS301	0.260	0.20	0.30
#7	2023Dec_03	Cupped	0.8	0.5	SUS301	0.260	0.20	0.50

Supplementary Table 1. (cont.)

Exp ID					
Run#	t/a or t/g	Gauge length of rad. col. (mm)	Ice VII prepared through low-T	Refined structural parameter	P_{max} (GPa)
#1	t/a	1.07	no	-	45.0
#2	t/a	1.07	no	-	78.9
#3	t/a	1.07	no	-	102
#4	t/a	0.5	yes	x(D), U(D), U(O)	39.2
#5	t/a	0.5	yes	x(D), U_{ij}(D)*	106
#6	t/g	0.5	yes	x(D)	48.2
#7	t/a	0.5	yes	x(D), U(D), U(O)	67.5

*Anisotropic ADPs (U_{ij}) for deuterium were refined only for the data taken at 103 GPa (Exp#79122-79123), see details in Supplementary information 6.

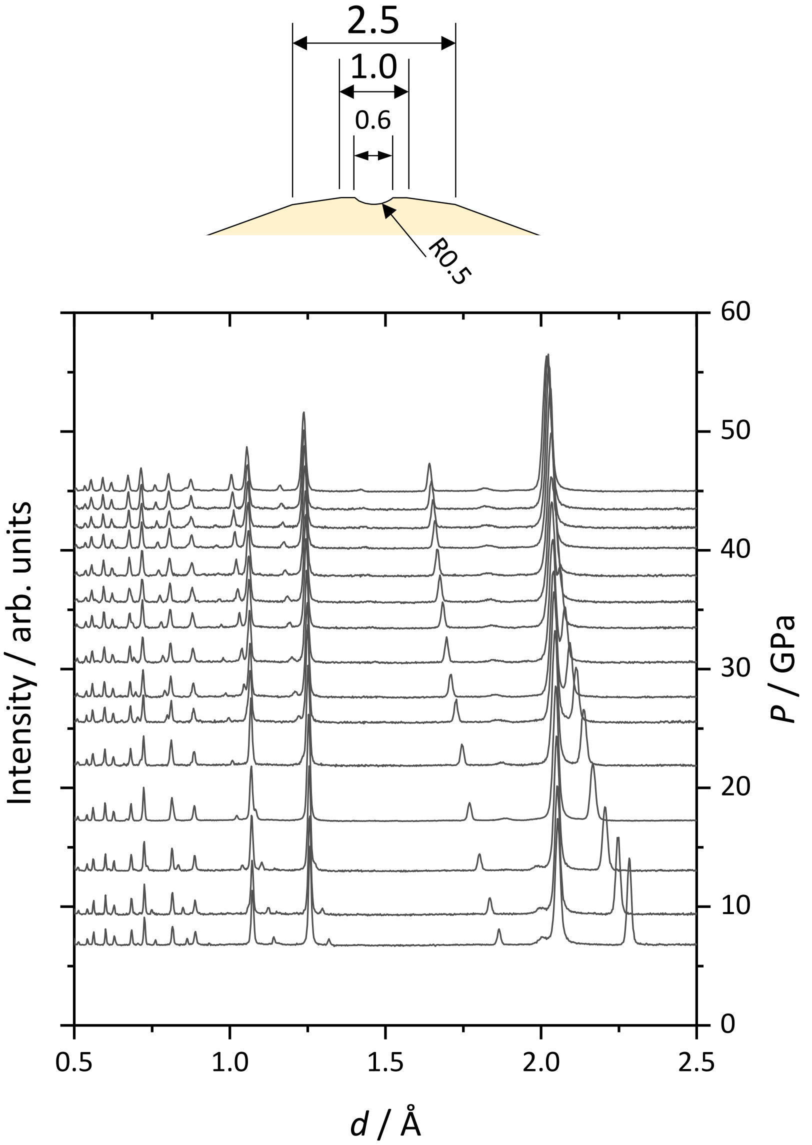

Supplementary Figure 1. Powder neutron diffraction patterns of ice VII obtained with the nano polycrystal diamond anvil cell (NPDAC) in run #1. Diffraction patterns are shifted such that they cross the right hand vertical axis at their measurement pressures. The shape of the anvil culets is also drawn on the top. R means the radius of the cup.

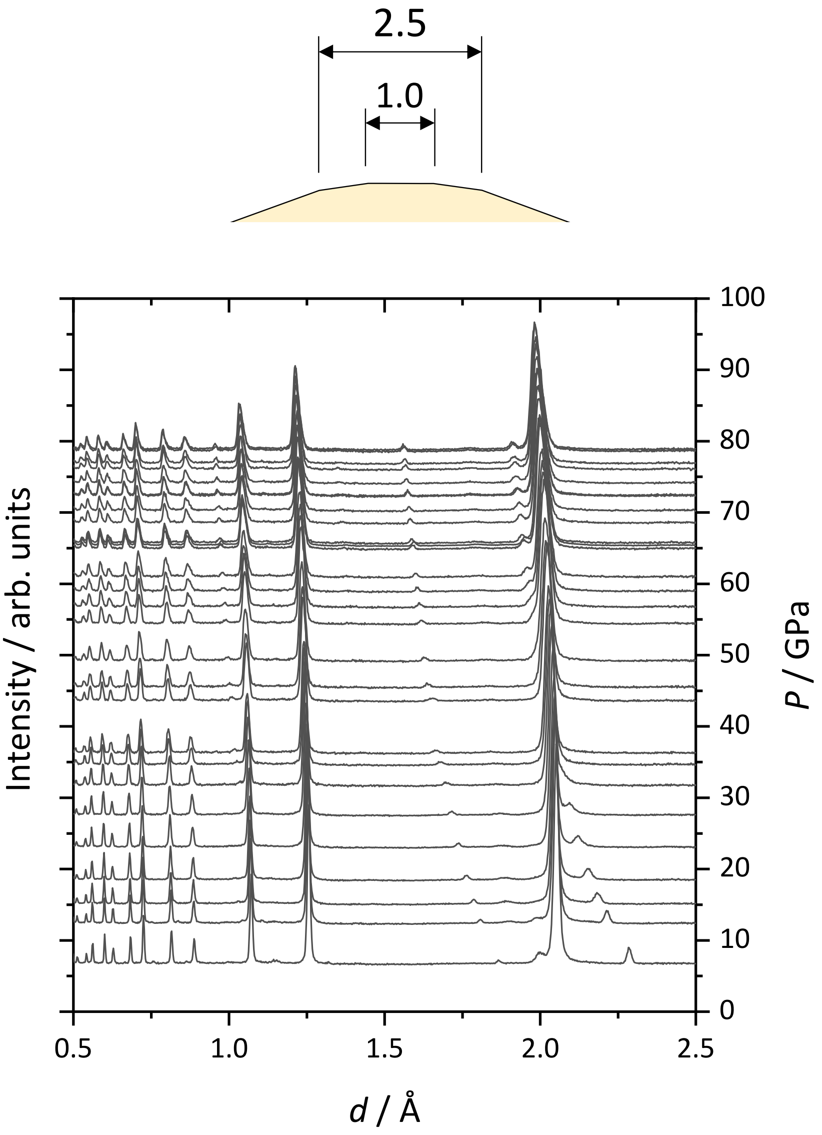

Supplementary Figure 2. Powder neutron diffraction patterns of ice VII obtained with the NPDAC in run #2. Diffraction patterns are shifted such that they cross the right hand vertical axis at their measurement pressures. The shape of the anvil culets is also drawn on the top.

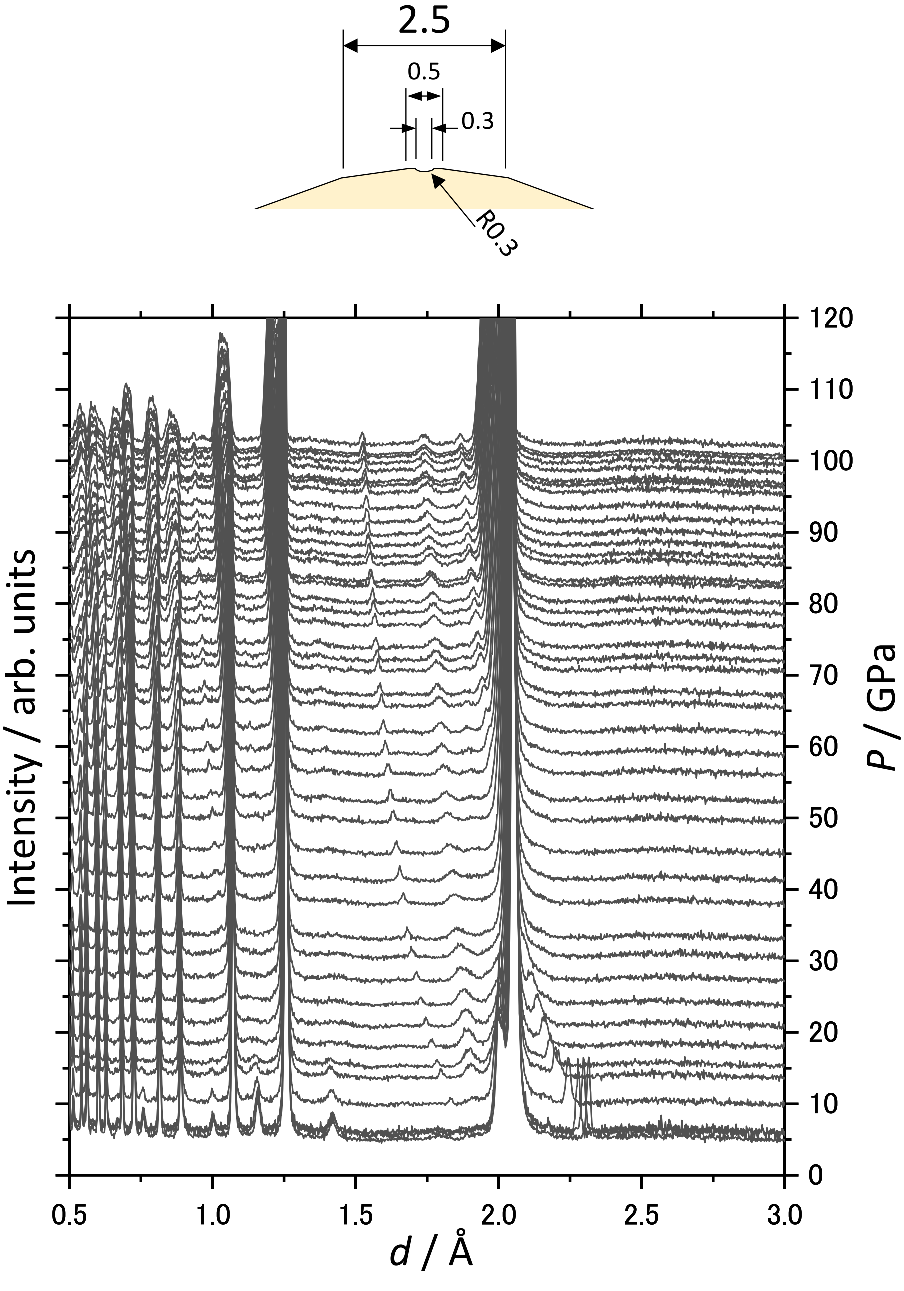

Supplementary Figure 3. Powder neutron diffraction patterns of ice VII obtained with the NPDAC in run #3. Diffraction patterns are shifted such that they cross the right hand vertical axis at their measurement pressures. The shape of the anvil culets is also drawn on the top. R means the radius of the cup.

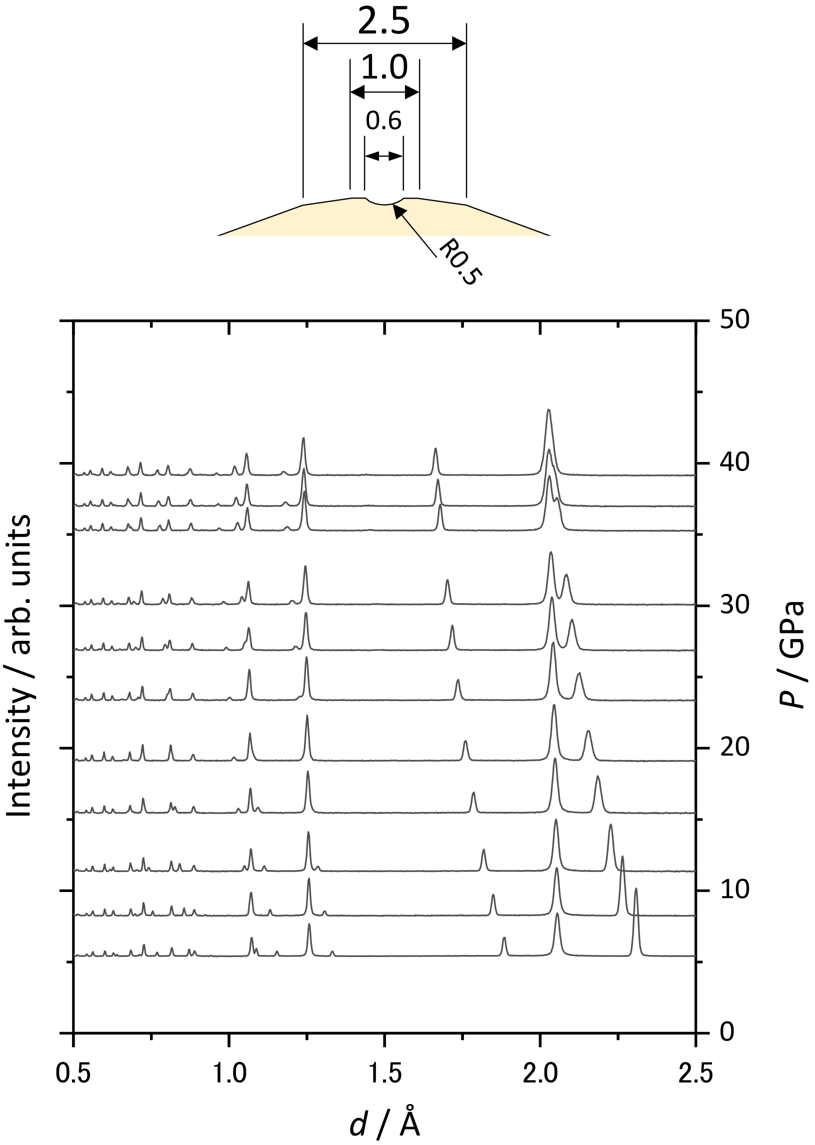

Supplementary Figure 4. Powder neutron diffraction patterns of ice VII obtained with the NPDAC in run #4. Diffraction patterns are shifted such that they cross the right hand vertical axis at their measurement pressures. The shape of the anvil culets is also drawn on the top. R means the radius of the cup.

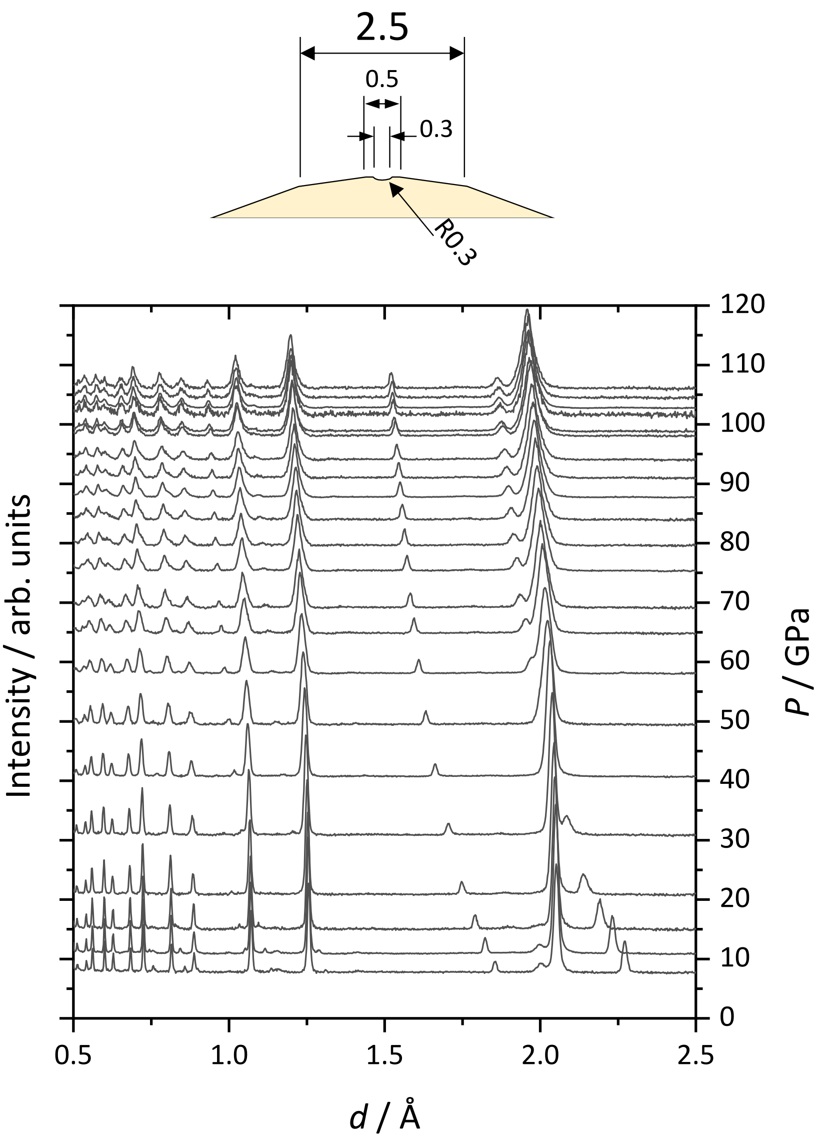

Supplementary Figure 5. Powder neutron diffraction patterns of ice VII obtained with the NPDAC in run #5. Diffraction patterns are shifted such that they cross the right hand vertical axis at their measurement pressures. The shape of the anvil culets is also drawn on the top. R means the radius of the cup.

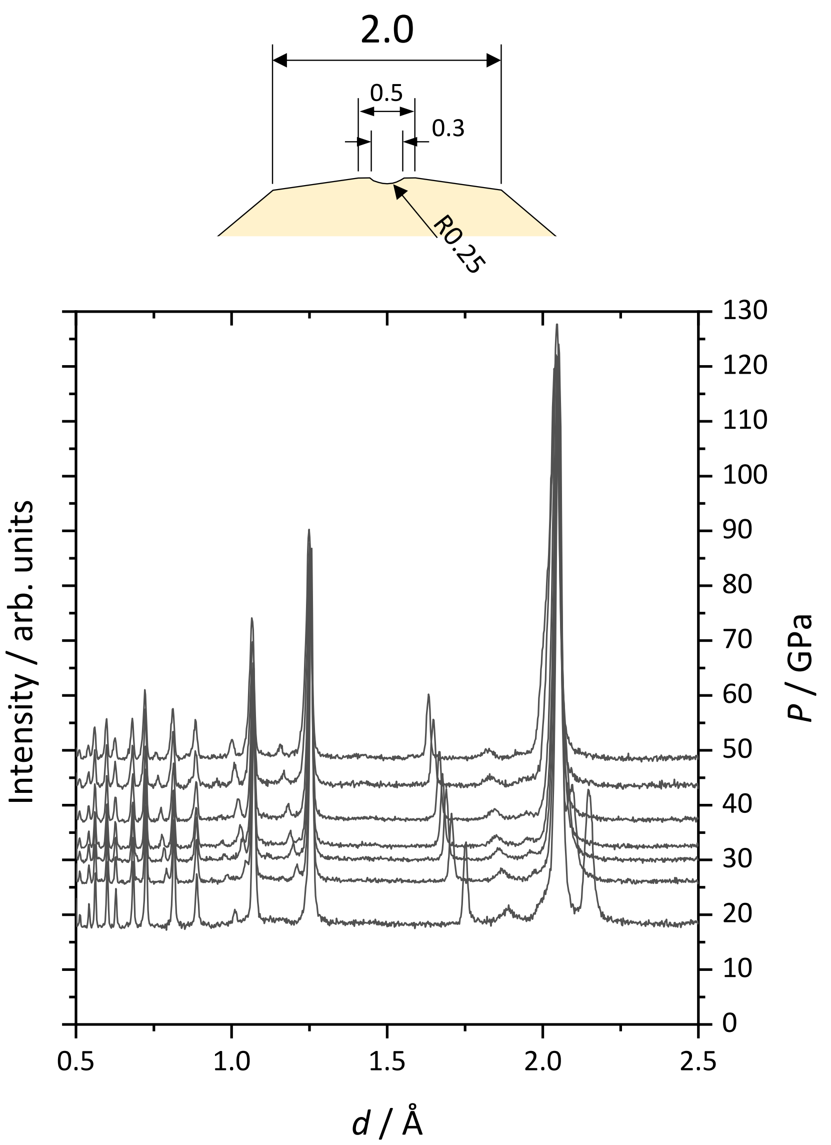

Supplementary Figure 6. Powder neutron diffraction patterns of ice VII obtained with the NPDAC in run #6. Diffraction patterns are shifted such that they cross the right hand vertical axis at their measurement pressures. The shape of the anvil culets is also drawn on the top. R means the radius of the cup.

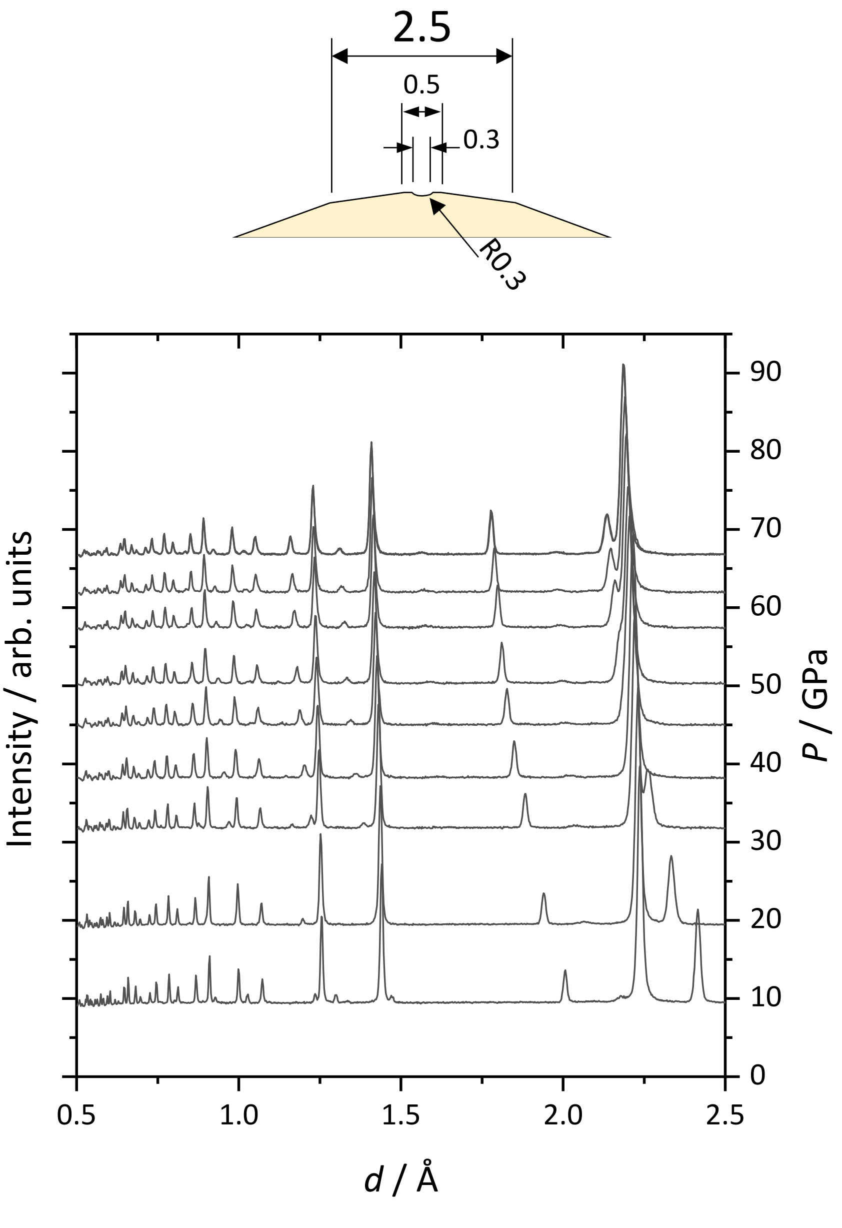

Supplementary Figure 7. Powder neutron diffraction patterns of ice VII obtained with the NPDAC in run #7. Diffraction patterns are shifted such that they cross the right hand vertical axis at their measurement pressures. The shape of the anvil culets is also drawn on the top. R means the radius of the cup.

Supplementary Table 2. Results of the Rietveld analyses for the dataset #1, and peak widths per *d*-spacing (*Δd/d*) for 110 and 111.

† These values are slightly different from the previously puslbished data[1] due to the re-analysis in this study.

Exp#	Load (tonne)	$a^\dagger$ (Å)	$V^\dagger$ (Å^3)	$P^\dagger$ (GPa)	$\Delta d/d$ 110	$\Delta d/d$ 111
59666	1.4	3.23218(13)	33.766(4)	6.77	0.00685(12)	0.00683(13)
59667	1.8	3.17956(8)	32.144(2)	9.43	0.00731(13)	0.00692(13)
59679	2.2	3.12127(10)	30.408(3)	13.0	0.00809(16)	0.00730(13)
59686	2.6	3.06597(5)	28.8205(14)	17.2	0.00885(18)	0.00754(19)
59688	3.0	3.01562(11)	27.424(3)	21.9	0.00891(19)	0.00736(18)
59689	3.4	2.98059(10)	26.479(3)	25.6	0.00908(19)	0.00753(19)
59690	3.8	2.9626(2)	26.004(6)	27.7	0.00998(19)	0.00763(16)
59691	4.2	2.93961(10)	25.402(3)	30.6	0.00996(15)	0.00745(16)
59698	4.6	2.91888(9)	24.868(2)	33.5	0.0093(2)	0.00748(16)
59699	5.0	2.90334(13)	24.473(3)	35.7	-	0.00762(16)
59700	5.4	2.88936(12)	24.122(3)	37.9	-	0.00754(16)
59701	5.8	2.8747(3)	23.757(6)	40.3	-	0.00759(14)
59702	6.2	2.86449(18)	23.504(4)	42.0	-	0.00786(16)
59703	6.6	2.85585(13)	23.292(3)	43.5	-	0.00784(16)
59704	7.0	2.84773(11)	23.094(3)	45.0	-	0.00774(15)

Supplementary Table 3. Results of the Rietveld analyses for the dataset #2, and peak widths per *d*-spacing (*Δd/d*) for 110 and 111.

Exp#	Load (tonne)	$a^\dagger$ (Å)	$V^\dagger$ (Å^3)	$P^\dagger$ (GPa)	$\Delta d/d$ 110	$\Delta d/d$ 111
59833	1.4	3.2317(3)	33.752(8)	6.79	0.00732(12)	0.0073(5)
59834	1.8	3.1309(3)	30.689(8)	12.4	0.0087(2)	0.0079(3)
59835	2.2	3.0923(2)	29.570(6)	15.1	0.0120(5)	0.0081(3)
59836	2.6	3.0508(5)	28.395(15)	18.5	0.0143(4)	0.0103(5)
59837	3.0	3.0035(3)	27.095(9)	23.1	0.0192(4)	0.0101(7)
59838	3.4	2.9636(11)	26.03(3)	27.6	0.0085(8)	0.0110(7)
59847	3.8	2.9317(10)	25.20(2)	31.7	-	0.0151(12)
59848	4.2	2.9099(5)	24.639(13)	34.8	-	0.0138(10)
59849	4.6	2.8994(9)	24.37(2)	36.3	-	0.0138(11)
59850	5.0	2.8554(6)	23.282(15)	43.6	-	0.0167(14)
59851	5.4	2.8449(7)	23.024(18)	45.5	-	0.0111(9)
59852	5.8	2.8256(8)	22.56(2)	49.2	-	0.0105(8)
59853	6.2	2.8005(15)	21.96(4)	54.4	-	0.0103(8)
59854	6.6	2.7898(7)	21.714(17)	56.8	-	0.0099(8)
59863	7.0	2.7803(8)	21.491(18)	59.0	-	0.0077(5)
59864	7.4	2.7715(4)	21.289(8)	61.1	0.0156(7)	0.0088(5)
59866	7.8	2.7542(4)	20.892(9)	65.4	0.0174(9)	0.0088(3)
59867	8.2	2.7557(4)	20.925(9)	65.1	0.0175(11)	0.0090(5)
59868	8.6	2.7526(5)	20.856(12)	65.9	0.0156(10)	0.0078(5)
59869	9.0	2.7423(4)	20.623(10)	68.6	0.0168(14)	0.0074(5)
59870	9.4	2.7363(4)	20.488(9)	70.2	0.0146(9)	0.0079(5)
59871	9.8	2.7286(4)	20.316(9)	72.4	0.0166(14)	0.0081(5)
59880	10.2	2.7282(4)	20.307(10)	72.5	0.0158(12)	0.0078(5)
59881	10.6	2.7221(4)	20.171(9)	74.2	0.0175(17)	0.0079(5)
59882	11.0	2.7156(6)	20.025(14)	76.2	0.0137(10)	0.0083(5)
59883	11.4	2.7129(4)	19.967(9)	76.9	0.0152(18)	0.0078(5)
59884	11.8	2.7074(5)	19.845(10)	78.6	0.0126(15)	0.0095(6)
59885	12.2	2.7064(4)	19.824(9)	78.9	0.0102(10)	0.0086(5)

† These values are slightly different from the previously puslbished data[1] due to the re-analysis in this study.

Supplementary Table 4. Results of the Rietveld analyses for the dataset #3, and peak widths per *d*-spacing ($\Delta d/d$) for 110 and 111.

Exp#	Load (tonne)	*a* (Å)	*V* (Å^3)	*P* (GPa)	$\Delta d/d$ 110	$\Delta d/d$ 111
69944	0.4	3.2744(2)	35.109(7)	4.97	0.00641(10)	-
69945	0.6	3.250(5)	34.31(15)	6.00	0.00645(15)	-
69946	0.8	3.2228(3)	33.473(9)	7.21	0.0072(3)	-
69947	1.2	3.174(4)	31.97(11)	9.76	0.0078(3)	0.0076(8)
69948	1.6	3.1174(4)	30.297(10)	13.3	0.0101(7)	0.0083(6)
69949	1.8	3.0808(4)	29.241(11)	16.0	0.0103(8)	0.0081(7)
69950	2.0	3.0543(7)	28.49(2)	18.2	0.0114(5)	0.0088(8)
69951	2.2	3.025(12)	27.7(3)	20.9	0.0131(11)	0.0078(6)
69952	2.4	2.9910(12)	26.76(3)	24.4	0.0136(17)	0.0094(9)
69953	2.6	2.9638(19)	26.04(5)	27.6	-	0.0086(8)
69962	2.8	2.9526(16)	25.74(4)	28.9	-	0.0110(10)
69963	3.0	2.909(3)	24.63(8)	34.8	-	0.0104(8)
69964	3.2	2.885(3)	24.01(7)	38.6	-	0.0117(8)
69965	3.4	2.871(13)	23.7(3)	40.9	-	0.0101(7)
69966	3.6	2.848(8)	23.1(2)	44.9	-	0.0108(7)
69967	3.8	2.825(11)	22.5(3)	49.3	-	0.0099(9)
69968	4.0	2.812(13)	22.2(3)	52.0	-	0.0085(6)
69969	4.2	2.795(3)	21.84(7)	55.6	-	0.0103(8)
69970	4.4	2.779(5)	21.47(13)	59.2	-	0.0087(5)
69971	4.6	2.7688(4)	21.225(9)	61.8	-	0.0087(6)
69972	4.8	2.7505(9)	20.81(2)	66.4	-	0.0074(5)
69973	5.0	2.7444(6)	20.670(14)	68.0	0.0125(9)	0.0094(7)
69974	5.2	2.7375(11)	20.52(2)	69.9	0.0121(9)	0.0078(6)
69975	5.4	2.7296(13)	20.34(3)	72.1	0.0153(18)	0.0088(7)
69976	5.6	2.720(10)	20.1(2)	74.9	0.0133(16)	0.0087(7)
69977	5.8	2.713(3)	19.97(6)	76.9	0.0102(12)	0.0082(5)
69978	6.0	2.7003(12)	19.69(3)	80.8	0.0074(8)	0.0086(6)
69979	6.2	2.7054(13)	19.80(3)	79.2	0.0095(8)	0.0079(5)
69980	6.4	2.691(4)	19.48(9)	83.9	0.0088(8)	0.0080(6)
69981	6.6	2.6856(10)	19.37(2)	85.6	0.0115(10)	0.0083(5)
69982	6.8	2.6884(6)	19.430(12)	84.7	0.0113(10)	0.0094(7)
69983	7.0	2.680(3)	19.26(6)	87.3	0.0089(7)	0.0074(5)
69984	7.2	2.6779(5)	19.204(10)	88.2	0.0090(9)	0.0084(7)
69985	7.4	2.676(5)	19.15(11)	89.0	0.0097(10)	0.0078(6)
69986	7.6	2.6693(6)	19.019(13)	91.2	0.0092(8)	0.0083(5)
69987	7.8	2.6693(5)	19.020(10)	91.2	0.0105(8)	0.0079(5)
69988	8.0	2.6591(17)	18.80(4)	94.9	0.0103(9)	0.0086(7)
69989	8.2	2.6616(4)	18.855(9)	94.0	0.0106(11)	0.0089(7)
69990	8.4	2.65(5)	18.7(10)	96.7	0.0114(13)	0.0078(6)
69991	8.6	2.6499(4)	18.607(9)	98.3	0.0091(10)	0.0089(6)
69992	8.8	2.6461(6)	18.528(12)	99.8	0.0087(11)	0.0088(6)
69993	9.0	2.6417(10)	18.44(2)	101	0.0101(5)	0.0085(4)
69995	9.2	2.6418(6)	18.437(12)	101	0.0115(13)	0.0085(6)
69996	9.4	2.6393(4)	18.385(8)	102	0.0098(9)	0.0090(7)

Supplementary Table 5. Results of the Rietveld analyses for the dataset #4, and peak widths per *d*-spacing (*Δd/d*) for 110 and 111.

Exp#	Load (tonne)	*a* (Å)	*V* ($Å^3$)	*P* (GPa)	*x* (D) (-)	*U* (D) ($Å^2$)	*U* (O) ($Å^2$)	*d* (O-D) (Å)	*d* (D…O) (Å)	*d* (D…D) (Å)	*Δd* /*d* 110	*Δd* /*d* 111
78829	1.0	3.2637(3)	34.765(10)	5.40	0.4124(3)	0.0262(7)	0.0155(5)	0.9179(16)	1.9085(16)	0.991(3)	0.00678(3)	0.00659(4)
78831	1.5	3.2022(4)	32.837(13)	8.22	0.4175(4)	0.0236(11)	0.0135(7)	0.929(2)	1.844(2)	0.915(5)	0.00712(3)	0.00655(4)
78839	2.0	3.1471(5)	31.170(14)	11.3	0.4221(4)	0.0222(9)	0.0109(7)	0.938(2)	1.787(2)	0.849(4)	0.00840(4)	0.00719(5)
78840	2.5	3.0886(5)	29.463(15)	15.4	0.4253(4)	0.0199(9)	0.0071(7)	0.938(2)	1.737(2)	0.799(4)	0.01003(7)	0.00774(5)
78841	3.0	3.0446(6)	28.221(16)	19.1	0.4295(4)	0.0203(9)	0.0117(12)	0.947(2)	1.690(2)	0.744(4)	0.01120(7)	0.00821(5)
78842	3.5	3.0013(6)	27.036(16)	23.3	0.4319(4)	0.0199(9)	0.0130(9)	0.945(2)	1.654(2)	0.708(4)	0.01157(9)	0.00819(6)
78850	4.0	2.9694(5)	26.181(14)	26.9	0.4351(4)	0.0197(8)	0.0106(6)	0.952(2)	1.619(2)	0.667(4)	0.01107(10)	0.00782(5)
78851	4.5	2.9437(5)	25.507(14)	30.1	0.4367(4)	0.0183(8)	0.0093(6)	0.952(2)	1.597(2)	0.645(4)	0.01171(15)	0.00800(4)
78852	5.5	2.9063(7)	24.547(17)	35.3	0.4397(4)	0.0171(8)	0.0089(6)	0.955(2)	1.562(2)	0.607(4)	0.0109(2)	0.00772(4)
78864	6.0	2.8952(8)	24.27(2)	37.0	0.4413(5)	0.0172(9)	0.0079(6)	0.959(2)	1.548(3)	0.589(5)	-	0.00769(4)
78885	6.5	2.8814(9)	23.92(2)	39.2	0.4428(5)	0.0168(9)	0.0089(6)	0.962(3)	1.533(3)	0.571(5)	-	0.00779(4)

Supplementary Table 6. Results of the Rietveld analyses for the dataset #5, and peak widths per *d*-spacing (*Δd/d*) for 110 and 111.

Exp#	Load (tonne)	*a* (Å)	*V* ($Å^3$)	*P* (GPa)	*x* (D) (-)	*U* (D)* ($Å^2$)	*U* (O)* ($Å^2$)	*d* (O-D) (Å)	*d* (D…O) (Å)	*d* (D…D) (Å)	*Δd* /*d* 110	*Δd* /*d* 111
79064	1.0	3.21327(16)	33.177(5)	7.67	0.4195(8)	0.0243	0.0147	0.943(4)	1.839(4)	0.896(9)	0.00751(10)	0.00709(16)
79074	1.5	3.1548(8)	31.40(2)	10.9	0.4223(5)	0.0228	0.0128	0.942(3)	1.791(3)	0.849(6)	0.00835(8)	0.00733(10)
79075	2.0	3.0950(2)	29.647(7)	14.9	0.4225(9)	0.0214	0.0109	0.925(5)	1.756(5)	0.831(10)	0.01032(17)	0.0078(3)
79085-86	2.5	3.0236(3)	27.643(7)	21.1	0.4279(10)	0.0197	0.0088	0.932(5)	1.687(5)	0.755(10)	0.0123(3)	0.0085(2)
79087	3.0	2.9379(3)	25.358(8)	30.9	0.4295(10)	0.0181	0.0069	0.914(5)	1.631(5)	0.717(10)	0.0174(6)	0.0095(2)
79088-89	3.5	2.8585(3)	23.357(7)	43.0	0.4343(12)	0.0171	0.0060	0.913(6)	1.563(6)	0.651(12)	-	0.00860(16)
79099	4.0	2.8247(3)	22.537(8)	49.4	0.4479(18)	0.0167	0.0055	0.968(9)	1.478(9)	0.510(18)	-	0.0085(2)
79100	4.5	2.7840(3)	21.577(6)	58.2	0.4504(12)	0.0164	0.0052	0.966(6)	1.445(6)	0.479(12)	0.0127(4)	0.00907(10)
79101	5.0	2.7563(3)	20.940(7)	64.9	0.4540(16)	0.0163	0.0051	0.974(8)	1.413(8)	0.439(16)	0.0126(6)	0.00898(17)
79111	5.5	2.7403(3)	20.578(7)	69.1	0.464(2)	0.0162	0.0051	1.014(9)	1.359(9)	0.345(19)	0.0096(7)	0.00910(17)
79112-13	6.0	2.7186(3)	20.092(6)	75.3	0.4634(17)	0.0161	0.0050	1.005(8)	1.349(8)	0.344(16)	0.0103(4)	0.00907(13)
79114	6.5	2.7036(3)	19.763(7)	79.8	0.469(2)	0.0161	0.0050	1.026(11)	1.315(11)	0.29(2)	0.0115(5)	0.00878(18)
79115	7.0	2.6907(3)	19.481(7)	83.9	0.471(2)	0.0161	0.0050	1.031(12)	1.300(12)	0.27(2)	0.0117(4)	0.00950(19)
79116	7.5	2.6792(3)	19.232(5)	87.7	0.472(2)	0.0161	0.0050	1.032(10)	1.288(10)	0.257(19)	0.0127(2)	0.00913(10)
79117	8.0	2.6704(3)	19.043(7)	90.8	0.470(2)	0.0161	0.0050	1.018(11)	1.294(11)	0.28(2)	0.0123(4)	0.00881(17)
79118	8.5	2.6615(3)	18.853(7)	94.0	0.477(3)	0.0160	0.0050	1.047(15)	1.258(15)	0.21(3)	0.0137(6)	0.00924(19)
79119	9.0	2.6509(4)	18.628(7)	97.9	0.479(4)	0.0160	0.0050	1.053(17)	1.242(17)	0.19(3)	0.0144(4)	0.0092(2)
79120	9.5	2.6486(3)	18.579(7)	98.8	0.481(4)	0.0160	0.0050	1.058(17)	1.236(17)	0.18(3)	0.0133(5)	0.0094(2)
79121	10.0	2.6420(7)	18.441(15)	101	0.482(9)	0.0160	0.0050	1.06(4)	1.23(4)	0.17(8)	0.0125(10)	0.0085(4)
79122-23	10.2	2.6383(3)	18.365(5)	103	0.484(4)	0.0160	0.0050	1.070(17)	1.215(17)	0.15(3)	0.0136(2)	0.00918(11)
79124	10.5	2.6340(4)	18.275(8)	105	0.488(7)	0.0160	0.0050	1.08(3)	1.20(3)	0.11(6)	0.0136(8)	0.0094(2)
79125	11.0	2.6299(4)	18.189(9)	106	0.484(6)	0.0160	0.0050	1.07(3)	1.21(3)	0.15(5)	0.0124(5)	0.0094(3)

*Fixed to the values from a fitted function to the dataset of runs #4 and #7 as shown in Fig. 3.

Supplementary Table 7. Results of the Rietveld analyses for the dataset #6, and peak widths per *d*-spacing (*Δd/d*) for 110 and 111.

Exp#	Load (tonne)	*a* (Å)	*V* ($Å^3$)	*P* (GPa)	*x* (D) (-)	*U* (D)* ($Å^2$)	*U* (O)* ($Å^2$)	*d* (O-D) (Å)	*d* (D…O) (Å)	*d* (D…D) (Å)	*Δd* /*d* 110	*Δd* /*d* 111
87495	1.0	3.0503(3)	28.380(7)	18.6	0.4274(9)	0.0203	0.0096	0.937(5)	1.704(5)	0.767(9)	0.0134(2)	0.00900(17)
87497-87498	1.4	2.9760(2)	26.357(6)	26.1	0.4341(8)	0.0187	0.0077	0.949(4)	1.628(4)	0.679(8)	0.0156(6)	0.00891(14)
87508	1.6	2.9256(2)	25.041(6)	32.5	0.4343(8)	0.0179	0.0067	0.934(4)	1.600(4)	0.666(8)	-	0.00872(14)
87509	1.8	2.9015(3)	24.427(7)	36.0	0.4360(10)	0.0175	0.0063	0.935(5)	1.578(5)	0.644(10)	-	0.0087(2)
87510	2.0	2.8904(3)	24.148(8)	37.7	0.4376(12)	0.0174	0.0062	0.939(6)	1.564(6)	0.624(12)	-	0.0086(2)
87522	2.2	2.8559(3)	23.292(8)	43.5	0.4411(13)	0.0170	0.0058	0.945(6)	1.528(6)	0.583(13)	-	0.0088(2)
87524	2.4	2.8304(3)	22.675(8)	48.3	0.4415(13)	0.0167	0.0055	0.939(7)	1.512(7)	0.574(13)	-	0.0083(2)

*Fixed to the values from a fitted function to the dataset of runs #4 and #7 as shown in Fig. 3.

Supplementary Table 8. Results of the Rietveld analyses for the dataset #7, and peak widths per *d*-spacing (*Δd/d*) for 110 and 111.

Exp#	Load (tonne)	*a* (Å)	*V* ($Å^3$)	*P* (GPa)	*x* (D) (-)	*U* (D) ($Å^2$)	*U* (O) ($Å^2$)	*d* (O-D) (Å)	*d* (D…O) (Å)	*d* (D…D) (Å)	*Δd* /*d* 110	*Δd* /*d* 111
91512	1.5	3.15006(10)	31.258(3)	11.17	0.4191(6)	0.0203(15)	0.0126(13)	0.923(3)	1.805(3)	0.882(6)	0.00828(5)	0.00725(7)
91521	2.5	3.02999(14)	27.818(4)	20.45	0.4247(7)	0.0192(11)	0.0128(18)	0.917(4)	1.707(4)	0.790(7)	0.01053(12)	0.00817(11)
91523	3.5	2.9281(10)	25.11(3)	32.2	0.4333(7)	0.0198(16)	0.0083(11)	0.930(4)	1.606(4)	0.676(7)	0.01080(5)	0.00866(12)
91524	4.5	2.88433(18)	23.996(5)	38.7	0.4367(8)	0.0187(11)	0.0058(9)	0.933(4)	1.565(4)	0.632(8)	-	0.00864(10)
91541	5.5	2.8451(2)	23.029(6)	45.5	0.4479(16)	0.0193(17)	0.0035(12)	0.975(8)	1.489(8)	0.513(16)	-	0.00894(10)
91542	6.5	2.8178(2)	22.374(5)	50.8	0.4493(15)	0.015(3)	0.0027(13)	0.973(7)	1.468(7)	0.495(15)	-	0.00869(10)
91543	7.5	2.78169(19)	21.524(4)	58.7	0.4596(18)	0.013(2)	0.0031(12)	1.010(9)	1.399(9)	0.389(18)	0.01352(18)	0.00880(9)
91544	8.5	2.76362(18)	21.107(4)	63.0	0.4665(18)	0.0175(14)	0.0025(10)	1.036(9)	1.357(9)	0.320(18)	0.01328(16)	0.00857(9)
91554	9.5	2.7484(2)	20.761(4)	66.9	0.468(2)	0.0162(15)	0.0029(12)	1.036(10)	1.344(10)	0.31(2)	0.01365(19)	0.00885(9)
91565	9.6	2.74642(14)	20.716(3)	67.5	0.4654(14)	0.0149(11)	0.0024(9)	1.025(7)	1.354(7)	0.329(13)	0.01311(14)	0.00867(7)

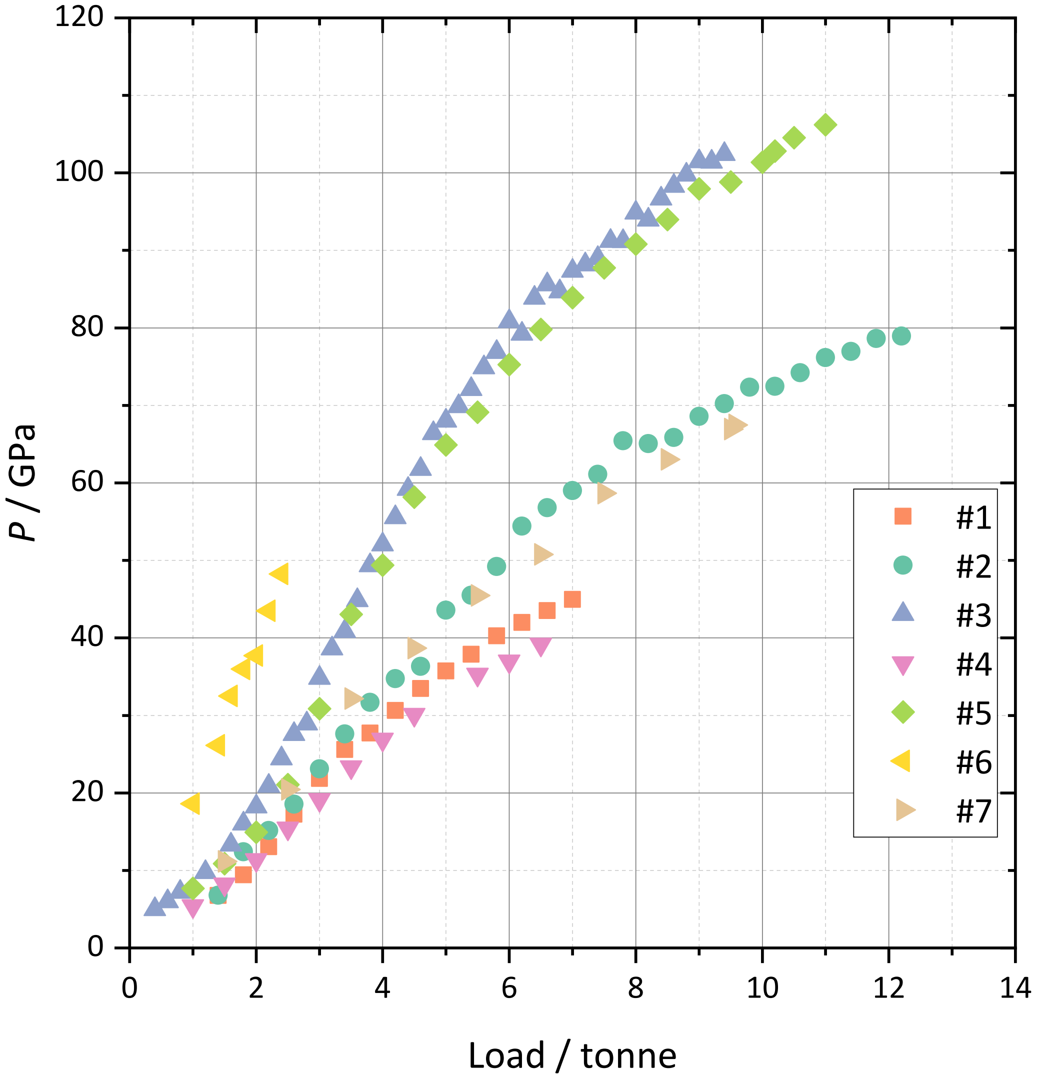

Supplementary Figure 8. Pressure generation as a function of load for respective runs.

2. Reliability and correlations of structural parameters

It is generally difficult to obtain absolute values for ADPs from data obtained under high pressure, since they may include unwanted scattering, in this case for instance, peaks from diamond anvils. As discussed in main text, we compare the isotropic ADPs (U_{iso}) for deuterium and oxygen with previously published ones (Supplementary Figure 9). Two datasets (#4 and #7) for individually loaded samples show good consistency, and consistent with previous data as shown in Supplementary Figure 8. The refined U_{iso} are scattered within approximately ±0.005 from the fitting by an exponential function.

$$U = a\exp(bP) + c \tag{2.1}$$

where U_0, a, b are empirical fitting parameters. These are a = 0.0131 Å^2, b = −0.0599 GPa^{-1}, and c = 0.0160 Å^2 for deuterium, and a = 0.0165 Å^2, b = −0.0696 GPa^{-1}, and c = 0.0049 Å^2 for oxygen, respectively.

In the Rietveld analyses, the ADPs were well converged in #4 owing to the relatively large sample size. The stability of the refinement can be assessed by the χ^2 map with varying possibly correlating parameters. Here we show one example that a positive correlation is found between atomic coordinate of deuterium, x(D), and the isotropic ADP, U(D) (Supplementary Figure 10). The clear minimum can be seen in the χ^2 map for the data taken in run #4, whereas the curvature at around the minimum for #5 is much shallow, showing the difficulty to obtain the absolute U_{iso} value in #5. Thus, we fixed the U_{iso} to the estimated value from eq. (2.1) in the refinements of the dataset for #5, and also for #6 by the same reason.

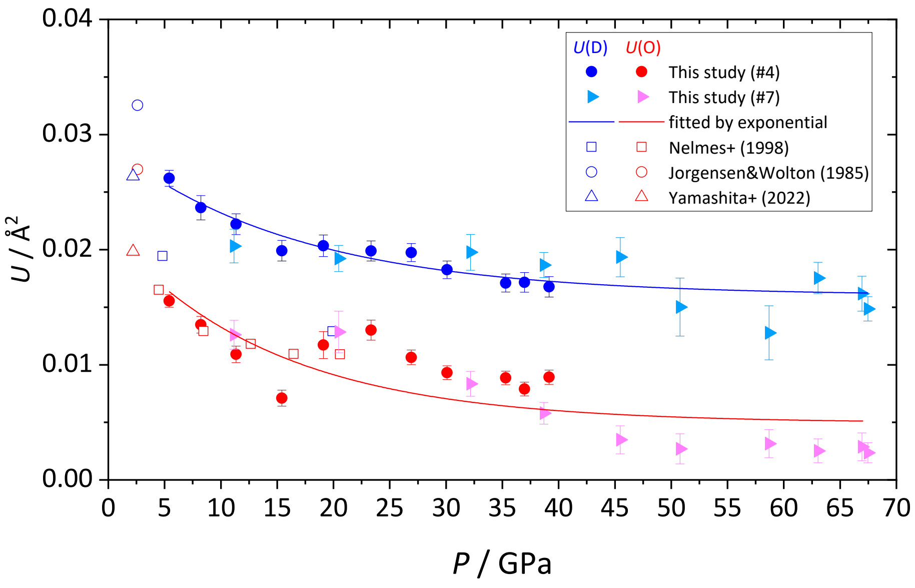

Supplementary Figure 9. Isotropic atomic displacement parameters (isotropic ADPs, U) for deuterium and oxygen atoms with estimated standard deviations shown as error bars obtained from the Rietveld analyses for run #4 with reported U values in previous studies (Jorgensen and Wolton[2], Nelmes et al.[3], and Yamashita et al.[4]). The U values were fitted with exponential functions shown by solid curves.

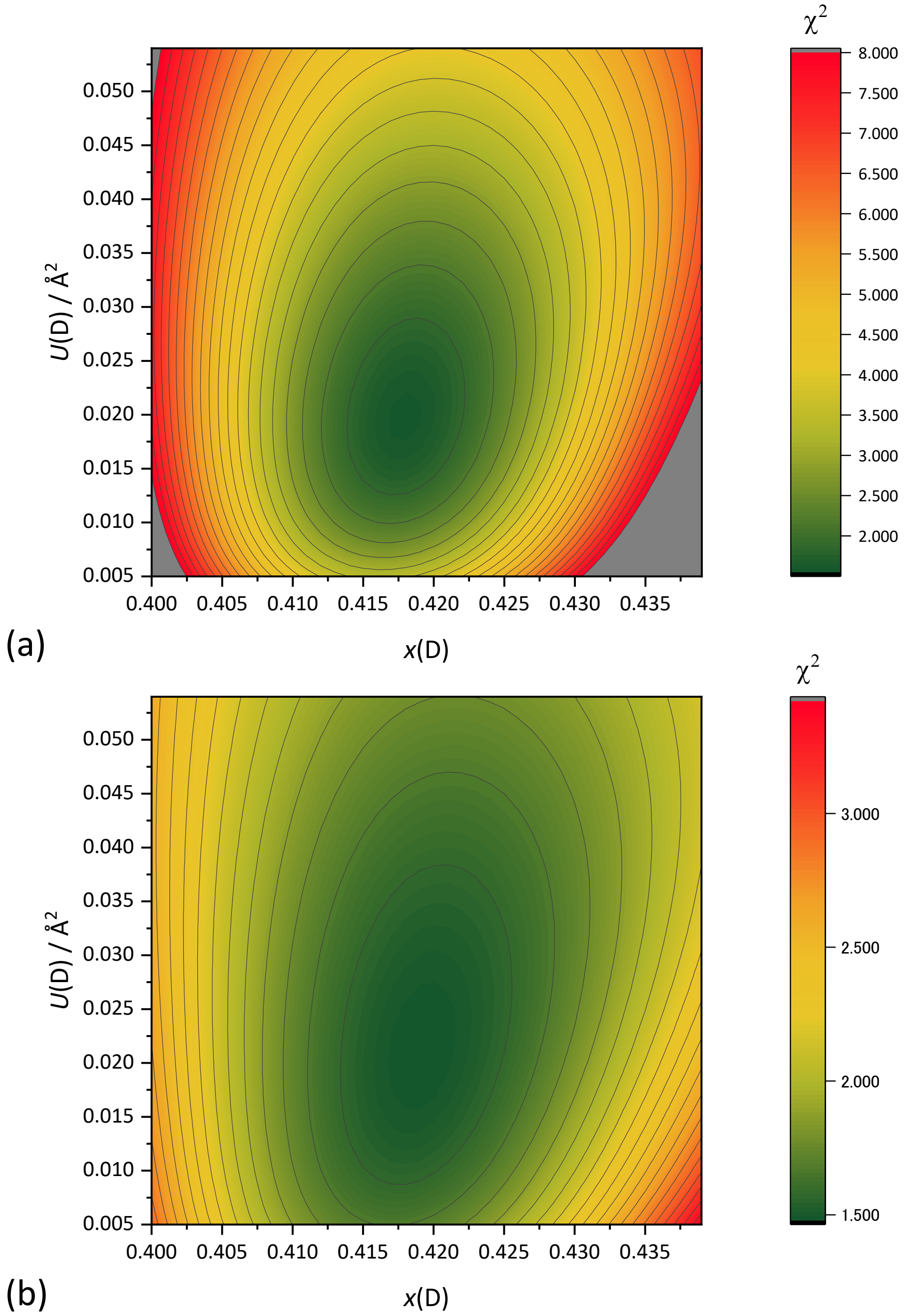

Supplementary Figure 10. The χ^2 values after the Rietveld analyses using GSAS[5] with varying atomic coordinate, *x*(D), and, isotropic ADP, *U*(D), for ice VII (a) at 8.22 GPa (run #4, exp #78831) and (b) at 7.67 GPa (run #5, exp #79064). The parameter range for *x*(D) is from 0.4 to 0.439 with 0.01 step, and that for *U*(D) is from 0.005 $Å^2$ to 0.054 $Å^2$ with 0.001 $Å^2$ step, so that 2000 χ^2 values in total are plotted as density maps.

3. A new NPDAC for through gasket geometry

The modified NPDAC is similar design to the original design[1] (see Supplementary Figure 11), the main difference is the tapered angle of the anvils to obtain the wider azimuth angle of scattered neutrons from original 20° to 40°. The NPD anvils have a bevel of 8° in the region within 2.5 mm in diameter, and the culet sizes and dip diameter in run #6 for through gasket geometry are 0.5 mm and 0.3 mm, respectively. The NPD anvils used in run #6 have 8.5 mm in diameter and 7 mm in height. The NPD anvils were provided with a supporting ring made of hardened steel (SNCM439), and set on the sintered diamond (SD in Supplementary Figure 8a). The modified NPDAC has no load generation mechanism as the original design, hence the forces for generating pressures are provided by a VX2 Paris Edinburgh press. The opening azimuth angle of the modified NPDAC is ±30° from the horizontal plane, which is slightly smaller than the detector coverage of the PLANET beamline (0 ± 34.6°)[6].

We also introduced a new incident beam collimator made of a metallic Gd plate with a hole (ϕ = 0.2 mm, t = 0.1 mm) attached to a hexagonal boron nitride (hBN) collimator with stepwise collimating (ϕ = 0.5 mm, l = 5.0 mm at the exit). The edge of the incident beam collimator was approx. 7 mm apart from the centre of the sample position. The incident beam collimator was attached onto a stepping-motor driven xz stage, and the position of the collimator was pre-adjusted by scanning using diffraction intensities from small iron sphere, and a NPDAC were adjusted so as to maximise the diffraction intensities from the sample.

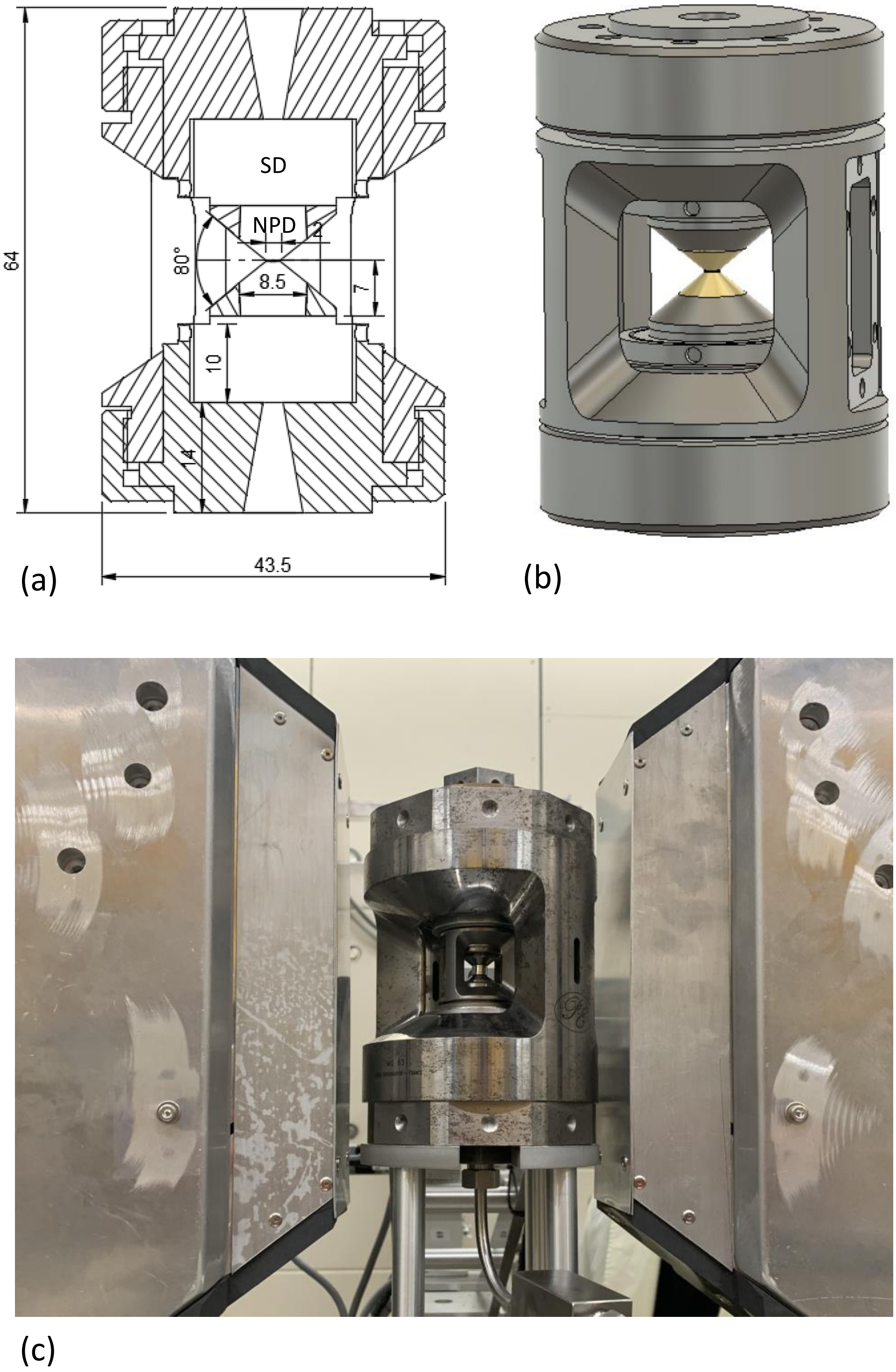

Supplementary Figure 11. Modified NPDAC for through gasket geometry. (a) Drawing of a section, (b) three-dimensional image, and (c) photo of the NPDAC set in Paris-Edinburgh press type VX2 on the PLANET diffractometer. SD in (a) means sintered diamond.

4. Attenuation corrections

Since the NPD anvils used in this study are polycrystalline material, conventional attenuation correction using intensities of incoherent scattering of vanadium can be applied to corrections for diffraction intensities, *i.e.*,

$$I_{cor.} = \frac{A_S^{-1}(I_S - I_E)}{A_V^{-1}(I_V - I_E)} \quad \text{(S4.1)}$$

where $I_{cor.}$ is the corrected diffraction intensity, I_S, I_E and I_V are intensities from sample (S), empty cell (E) and vanadium (V), respectively, A_s and A_V are attenuation factors for sample and vanadium, respectively. Because the scattering intensity from vanadium in the NPD cell was observed at ambient pressure, I_V should be corrected considering the attenuation of NPD anvils, which has significant pressure dependence. In order to correct the attenuation of anvils, we measured neutron transmission intensities through a pair of anvils with diffraction measurements at ambient pressure and also at high pressures as shown in Supplementary Figure 12 using a ^{3}He neutron monitor. The transmitted neutron intensities through anvils (I_{M2}) would be expressed as follows.

$$I_{M2}(\lambda, P) = E_M(\lambda)I_0(\lambda)\exp(-2\mu_d(\lambda, P)t_d) \quad \text{(S4.2)}$$

where $E_M(\lambda)$ is an efficiency of the neutron monitor as a function of wavelength (λ), $I_0(\lambda)$ is the intensity of incident neutron as a function of wavelength, $\mu_d(\lambda, P)$ is the attenuation coefficient of the diamond as a function of wavelength and pressure, and t_d is a thickness of diamonds. The ratio between the transmitted neutron intensities at ambient and high pressures gives the attenuation term of one diamond as follows:

$$\sqrt{\frac{I_{M2(\lambda,P)}}{I_{M2(\lambda,0)}}} = \exp(-\mu_d(\lambda, P)t_d - \mu_d(\lambda, 0)t_d) \quad \text{(S4.3)}$$

On the other hand, the observed scattering intensity from vanadium at ambient pressure $(I_V(\lambda, 0))$, and diffraction intensities from the sample would be described as follows.

$$I_V(\lambda, 0) = E_D(\lambda)I_0(\lambda)\Sigma_V \exp\left(-\mu_d(\lambda, 0)t_d - \mu_g(\lambda)t_g\right) + I_{bkg} \quad \text{(S4.4)}$$

$$I_S(\lambda, P) = E_D(\lambda)I_0(\lambda)\Sigma_S \exp\left(-\mu_d(\lambda, P)t_d - \mu_g(\lambda)t_g\right) + I_{bkg} \quad \text{(S4.5)}$$

where $E_D(\lambda)$ is an efficiency of the neutron detector as a function of wavelength, Σ_V and Σ_S are scattering cross sections of vanadium and sample (note that they are not symbols of summation), and I_{bkg} is the background intensities, which is approximated by I_E. Finally, the scattering intensity from vanadium under pressure can be estimated from the observable intensities as follows.

$$\begin{aligned} I_V(\lambda, P) &= E_D(\lambda)I_0(\lambda)\Sigma_V \exp\left(-\mu_d(\lambda, P)t_d - \mu_g(\lambda)t_g\right) + I_{bkg} \\ &= \left(I_V(\lambda, 0) - I_{bkg}\right)\sqrt{\frac{I_{M2}(\lambda, P)}{I_{M1}(\lambda, 0)}} + I_{bkg} \end{aligned} \quad \text{(S4.6)}$$

Here we suppose $I_{bkg} = I_E$, and the derived $I_V(\lambda, P)$ was used as I_V in eq. S4.1 to normalise the observed sample intensity.

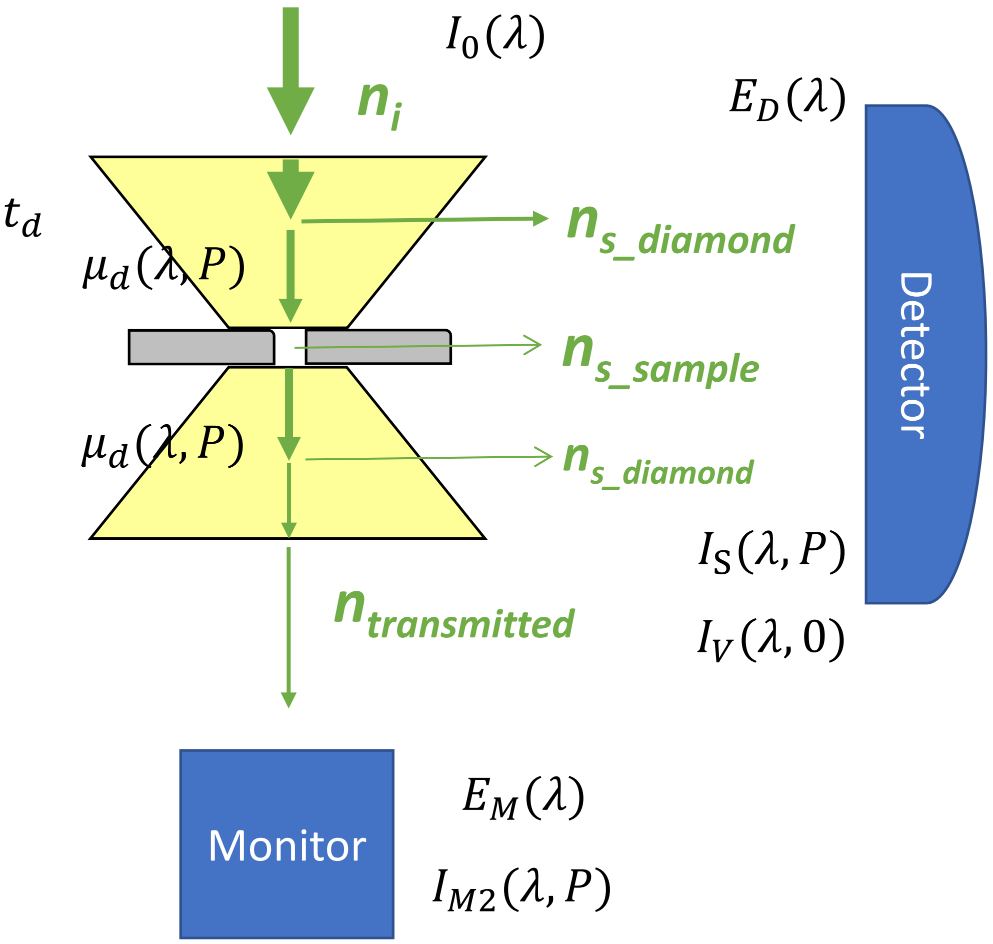

Supplementary Figure 12. Schematic illustration for attenuation corrections. Abbreviations, I : intensity, E: efficiency, μ : attenuation coefficient as functions of λ: wavelength, P: pressure, for S: sample, V: vanadium, d: diamond, g: gasket, M: monitor, D: detector as subscripted.

5. Anisotropic microstrain broadening

Stephens[7] described the microstrain broadening by a semi-empirical form, beginning with the expression for a reflection d-spacing as follows.

$$\frac{1}{d_{hkl}^2} = M_{hkl} = \alpha_1 h^2 + \alpha_2 k^2 + \alpha_3 l^2 + \alpha_4 kl + \alpha_5 hl + \alpha_6 hk \qquad \text{(S5.1)}$$

where $\alpha_i : i = 1, \ldots, 6$ are metric parameters of the reciprocal lattice. The microstrain broadening may be proportional to the variance of M_{hkl}, $\sigma^2(M_{hkl})$, and it is expressed by a variance-covariance matrix for α_i, C_{ij}.

$$\sigma^2(M_{hkl}) = \sum_{i,j} C_{ij} \frac{\partial M}{\partial \alpha_i} \frac{\partial M}{\partial \alpha_j} \qquad \text{(S5.2)}$$

Note that $\frac{\partial M}{\partial \alpha_1} = h^2, \frac{\partial M}{\partial \alpha_6} = hk, etc.$, the $\sigma^2(M_{hkl})$ can be written as

$$\sigma^2(M_{hkl}) = \sum_{HKL} S_{HKL} h^H k^K l^L \qquad (S5.3)$$

where S_{HKL} defined for $H + K + L = 4$. There are 15 parameters for S_{HKL} in the most general triclinic system, and even in the cubic system, two parameters, S_{400} and S_{220}, are remained as independent variable parameters (see more details in Stephens[7] for this derivation).

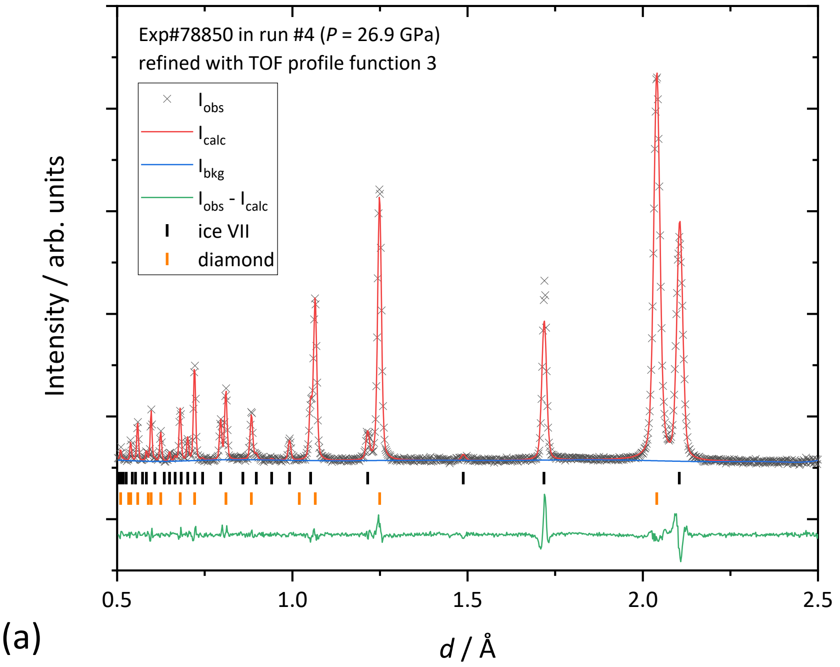

(a)

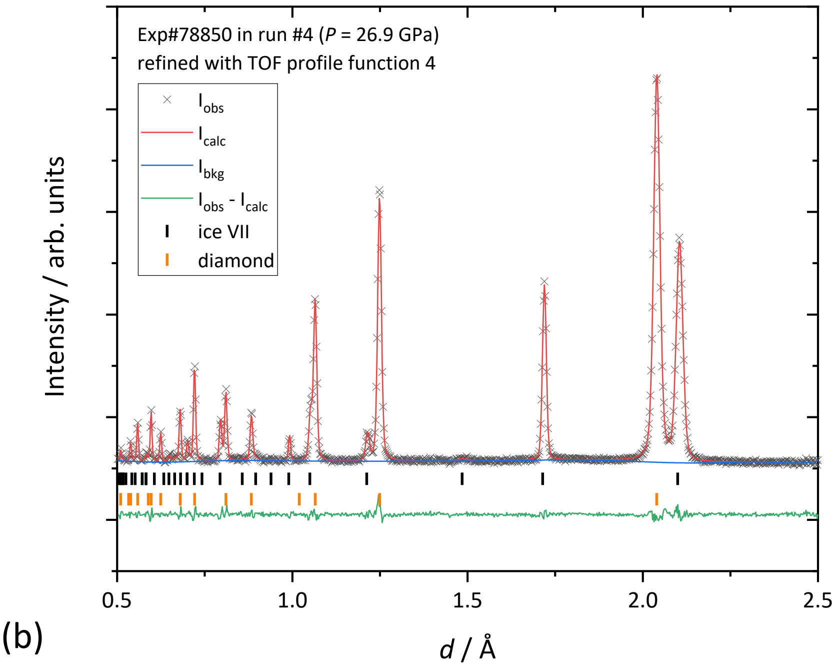

(b)

Supplementary Figure 13. Comparison of the Rietveld profile fitting between (a) TOF profile functions 3 and (b) TOF profile function 4, for the neutron diffraction data taken at 26.9 GPa in run #4. Anisotropic microstrain broadening is taken into account in the TOF4 profile function, but not in the TOF3 profile function.

6. Atomic distribution of deuterium

The eq. (2), the x-axis projection of probability density function of disordered deuterium at two sites with a distance, d, is rewritten here.

$$P_2(x) = \frac{1}{2(2\pi U)^{\frac{1}{2}}} \exp\left(-\frac{(x+d/2)^2}{2U}\right) + \frac{1}{2(2\pi U)^{\frac{1}{2}}} \exp\left(-\frac{(x-d/2)^2}{2U}\right) \tag{2}$$

The 2nd derivative of $P_2(x)$ is

$$\frac{d^2P_2(x)}{dx^2} = \frac{\exp\left(-\frac{(d+2x)^2}{8U}\right)}{8(2\pi U^5)^{\frac{1}{2}}}\left[d^2(1+\exp\frac{dx}{U}) - 4d(-1+\exp\frac{dx}{U})x - 4(1+\exp\frac{dx}{U})(U-x^2)\right] \tag{S6.1}$$

Then, the 2nd derivative of $P_2(x)$ at $x = 0$ is written as follows.

$$\left.\frac{d^2P_2(x)}{dx^2}\right|_{x=0} = \frac{\exp\left(-\frac{d^2}{8U}\right)}{4(2\pi U^5)^{\frac{1}{2}}}(d^2 - 4U) \tag{S6.2}$$

Here the conditions, $d > 0$ and $U > 0$, give $\frac{\exp\left(-\frac{d^2}{8U}\right)}{4(2\pi U^5)^{\frac{1}{2}}} > 0$, hence when $\left.\frac{d^2P_2(x)}{dx^2}\right|_{x=0} = 0$, the condition $d = 2\sqrt{U}$ will be given.

Anisotropic ADPs, U_{ij}, are constrained by the site symmetry to have two components, one parallel to the O-D direction, $U_{\parallel}$(D), and one perpendicular, $U_{\perp}$(D), in ice VII structure[3], such that the following conditions should be obeyed.

$$U_{11} = U_{22} = U_{33} = \frac{1}{3}U_{\parallel} + \frac{2}{3}U_{\perp}$$

$$U_{12} = U_{13} = U_{23} = \frac{1}{3}U_{\parallel} - \frac{1}{3}U_{\perp} \tag{S6.3}$$

Thus, the two components are given by anisotropic ADPs,

$$U_{\parallel} = U_{ii} + 2U_{ij}, U_{\perp} = U_{ii} - U_{ij} \tag{S6.4}$$

where $i \neq j$.

We have conducted the Rietveld analysis with varying anisotropic ADPs for the data taken at 103 GPa. In the refinement, we fixed the deuterium position to be x = 0.4999 with half occupancy instead of the completely centred position (x = 0.5) with full occupancy, since the anisotropic ADPs will be constrained by the site symmetry to be exactly spheric when x = 0.5. The χ^2 of the refinement based on the (pseudo) one-site model with the anisotropic ADPs is 7.104, whereas that based on the two-site model with the isotropic ADP is 7.095, so the two models give almost identical consistency to the obtained diffraction pattern taken at 103 GPa.

References

1. Komatsu, K. *et al.* Developments of nano-polycrystalline diamond anvil cells for neutron diffraction experiments. *High. Press. Res.* **40**, 184-193 (2020).
2. Jorgensen, J. D. & Worlton, T. G. Disordered structure of D_2O ice VII from in situ neutron powder diffraction. *J. Chem. Phys.* **83**, 329-333 (1985).
3. Nelmes, R. J. *et al.* Multisite Disordered Structure of Ice VII to 20 GPa. *Phys. Rev. Lett.* **81**, 2719-2722 (1998).
4. Yamashita, K. *et al.* Atomic distribution and local structure in ice VII from in situ neutron diffraction. *Proc. Nat. Acad. Sci.* **119**, e2208717119 (2022).
5. Larson, A. & Von Dreele, R. General Structure Analysis System (GSAS). *Los Alamos National Laboratory, Report LAUR-86-748* (2004).
6. Hattori, T. *et al.* Design and performance of high-pressure PLANET beamline at pulsed neutron source at J-PARC. *Nucl. Instr., Meth. Phys. Res. A* **780**, 55-67 (2015).
7. Stephens, P. Phenomenological model of anisotropic peak broadening in powder diffraction. *J. Appl. Crystallogr.* **32**, 281-289 (1999).
